# Utility of brief questionnaires of health-related quality of life (Airways Questionnaire 20 and Clinical COPD Questionnaire) to predict exacerbations in patients with asthma and COPD

**DOI:** 10.1186/1477-7525-11-85

**Published:** 2013-05-27

**Authors:** Marina Blanco-Aparicio, Isabel Vázquez, Salvador Pita-Fernández, Sonia Pértega-Diaz, Héctor Verea-Hernando

**Affiliations:** 1 Servicio de Neumología, Complejo Hospitalario Universitario A Coruña, A Coruña, Spain; 2 Departamento de Psicología Clínica y Psicobiología, Facultad de Psicología, Universidad de Santiago de Compostela, Santiago de Compostela, Spain; 3 Unidad de Epidemiología Clínica y Bioestadística, Complejo Hospitalario, Universitario A Coruña, A Coruña, Spain

**Keywords:** COPD, Asthma, Exacerbation, Risk factors, Emergency visits, Hospitalization, Health-related quality of life

## Abstract

**Background:**

There is some evidence that quality of life measured by long disease-specific questionnaires may predict exacerbations in asthma and COPD, however brief quality of life tools, such as the Airways Questionnaire 20 (AQ20) or the Clinical COPD Questionnaire (CCQ), have not yet been evaluated as predictors of hospital exacerbations.

**Objectives:**

To determine the ability of brief specific health-related quality of life (HRQoL) questionnaires (AQ20 and CCQ) to predict emergency department visits (ED) and hospitalizations in patients with asthma and COPD, and to compare them to longer disease-specific questionnaires, such as the St George´s Respiratory Questionnaire (SGRQ), the Chronic Respiratory Disease Questionnaire (CRQ) and the Asthma Quality of Life Questionnaire (AQLQ).

**Methods:**

We conducted a two-year prospective cohort study of 208 adult patients (108 asthma, 100 COPD). Baseline sociodemographic, clinical, functional and psychological variables were assessed. All patients completed the AQ20 and the SGRQ. COPD patients also completed the CCQ and the CRQ, while asthmatic patients completed the AQLQ. We registered all exacerbations that required ED or hospitalizations in the follow-up period. Differences between groups (zero ED visits or hospitalizations versus ≥ 1 ED visits or hospitalizations) were tested with Pearson´s X^2^ or Fisher´s exact test for categorical variables, ANOVA for normally distributed continuous variables, and Mann–Whitney U test for non-normally distributed variables. Logistic regression analyses were performed to estimate the predictive ability of each HRQoL questionnaire.

**Results:**

In the first year of follow-up, the AQ20 scores predicted both ED visits (OR: 1.19; p = .004; AUC 0.723) and hospitalizations (OR: 1.21; p = .04; AUC 0.759) for asthma patients, and the CCQ emerged as independent predictor of ED visits in COPD patients (OR: 1.06; p = .036; AUC 0.651), after adjusting for sociodemographic, clinical, and psychological variables. Among the longer disease-specific questionnaires, only the AQLQ emerged as predictor of ED visits in asthma patients (OR: 0.9; p = .002; AUC 0.727). In the second year of follow-up, none of HRQoL questionnaires predicted exacerbations.

**Conclusions:**

AQ20 predicts exacerbations in asthma and CCQ predicts ED visits in COPD in the first year of follow-up. Their predictive ability is similar to or even higher than that of longer disease-specific questionnaires.

## Background

Exacerbations occur commonly in patients with asthma and chronic obstructive pulmonary disease (COPD) resulting in clinical deterioration, accelerated lung function decline [1], increased of mortality [2] and worsening of health-related quality of life (HRQoL)[3]. This further leads to an increased number of required medical consultations, emergency department (ED) visits and hospitalizations [4], with the consequent increase of costs [5,6]. Therefore, it would be very important to identify patients with higher risk of exacerbations in order to take preventive measures.

Several sociodemographic, clinical and psychological factors are related to a higher risk of ED visits and hospitalizations, both in asthma [7,8] and COPD [9,10]. HRQoL has also been recently found to be related to the use of healthcare resources [11,12]. Among specific questionnaires, the St George´s Respiratory Questionnaire (SGRQ) [13] and the Asthma Quality of Life Questionnaire (AQLQ) [14] are related to likelihood of exacerbations in asthma, and the SGRQ has shown ability to predict admissions and readmissions in COPD patients [3,10,12].

Nevertheless, in spite of the proven utility of these questionnaires, their routine use in clinical practice is limited as they are long and time-consuming to complete. Brief quality of life tools, such as the Airways Questionnaire 20 (AQ20) [15] or the Clinical COPD Questionnaire (CCQ) [16], have not yet been evaluated as predictors of hospital exacerbations in patients with asthma and COPD.

Furthermore, the association between scores on HRQoL questionnaires and exacerbations has been estimated controlling for sociodemographic, clinical and functional variables [3,14,17], but very few studies have controlled for anxiety and depression. Both variables have been reported to be related to the use of healthcare resources [8,10] and HRQoL [18,19].

This study is aimed to 1) determine the ability of brief questionnaires AQ20 and CCQ to predict ED visits and hospitalizations in patients with asthma and COPD, controlling for the possible confounding effect of sociodemographic, clinical, functional and psychological variables; and 2) compare the predictive ability for exacerbations between these brief questionnaires and the longer disease-specific questionnaires such as SGRQ, Chronic Respiratory Disease Questionnaire (CRQ) and AQLQ.

## Material and methods

### Patients

A prospective non-intervention cohort study with a two-year follow-up was carried out in the University Hospital of A Coruña (Spain).

We selected a cohort of 208 patients (108 asthma and 100 COPD) with no associated comorbidity, who took part in the Spanish validation study of the AQ20 questionnaire. The asthma entry criteria were as follows: (a) diagnosis of asthma made by a respiratory physician; (b) increase greater than 12% in FEV_1_ or more than 20% in PEFR, following a dose of 200 ug salbutamol, or airway hyperresponsiveness, assessed by methacholine inhalation challenge test, observed on past evaluations; (c) age over 16; (d) smoking history of less than 20 pack-years; and (e) in smoking patients, FEV_1_/FVC ratio higher than 0.7 was required to exclude COPD.

The COPD entry criteria were: (a) diagnosis according to GOLD strategy [20], (b) post-bronchodilator FEV_1_/FVC ratio lower than 0.7 and FEV_1_ lower than 80% of the predicted value, (c) age over 40, (d) smoking history of 20 or more pack-years, and (e) no history suggestive of asthma.

Full details of the selection procedure have been already published [21].

### Measures

The AQ20 [15] is a specific questionnaire validated for patients with asthma or COPD. It includes 20 items with “yes” responses scored as 1, and “no” and “not applicable” scored as 0. The summary score ranges from 0 (best health) to 20 (worst health). It shows good reliability and validity [22,23].

The CCQ [16] is a COPD-specific questionnaire with 10 items answered on a seven-point Likert-type scale. It consists of three domains: Symptoms, Functional State and Mental State. The total score and the scores of the domains are the sum of scores given to each item, divided by the number of items. Thus, total score as well as the score on each of the three domains varies from 0 (best health) to 6 (worst health). The CCQ has shown good test-retest reliability, responsiveness and convergent and divergent validity [16].

The SGRQ [24] is a 50-item questionnaire specific for obstructive respiratory disease, which provides a total score and three component scores: Symptoms, Activity and Impact. Each score ranges from 0 (best health) to 100 (worst health). It has a good repeatability, it is correlated with a range of established measures of disease activity, and it responds to improvement and deterioration in the health of patients with chronic airflow limitation [24].

The CRQ [25] is a COPD-specific questionnaire that consists of 20 items comprising four domains: Dyspnea, Fatigue, Emotional Function and Mastery. Each domain is scored from 1 (worst quality of life) to 7 (best quality of life) and a total score can be calculated as the sum of scores on the four domains. It has shown good psychometric properties (reliability, validity and responsiveness) [25].

The AQLQ [26] is an asthma-specific questionnaire composed of 32 items which comprise four domains: Symptoms, Activities, Emotional Functioning and Environmental Stimuli. Each domain can be scored from 1 (worst health) to 7 (best health). A total score can be obtained as the sum of scores on the four domains. It has demonstrated good reliability, construct validity and responsiveness [27]. The Hospital Anxiety and Depression Scale (HADS) [28] has two 7-item subscales to measure anxiety and depression. Responses are rated on a 4-point Likert-type scale. Scores range from 0 to 21 for each subscale and a cut-off point of eleven identifies clinical disorders. It is a reliable and valid instrument for assessing anxiety and depression in medical patients [29].

The Spanish versions of all questionnaires were used [30-35] and good psychometric properties were demonstrated in all cases [20,31,32,34,36,37].

### Procedure

In the baseline assessment, we reviewed the clinical reports and carried out interviews with patients to collect information about sociodemographic variables (age, sex, age at diagnosis, smoking habit, educational and socioeconomical level, employment status, place of residence and marital status). A spirometry was carried out as described previously [21]. COPD patients also carried out the 6 minute walking test (6MWT) [38] and the BODE index was calculated on a 0 to 10 scale [39]. All patients completed the AQ20, SGRQ and HADS questionnaires. COPD patients also completed the CCQ and the CRQ, and asthmatic patients also filled in the AQLQ. We registered all exacerbations that required ED visits or hospitalizations, defined as an increase of respiratory symptoms that required a modification in treatment, during the two years after the baseline assessment. Data were obtained from the computer system and the review of clinical reports.

The study was approved by the Local Ethical Committee of Clinical Investigation (CEIC of Galicia: 2009/396) and written informed consent was obtained from all patients.

### Statistical Analysis

All results are presented as mean ± SD, or frequency and percentage. Patients were divided into two categories (zero ED visits or hospitalizations versus ≥ 1 ED visits or hospitalizations per year of follow-up).

Differences in sociodemographic, clinical, functional and psychological variables and in scores on HRQoL questionnaires between patients with and without exacerbations were tested with Pearson´s X^2^ or Fisher´s Exact test for categorical variables, ANOVA for normally distributed continuous variables, and Mann–Whitney U test for non-normally distributed variables.

Logistic regression analyses were conducted to estimate the ability of each questionnaire to predict the use of healthcare resources. Analyses were performed separately for those measures of HRQoL that showed differences in baseline scores between patients with and without exacerbations during follow-up. Age and sex were included as covariates in all models, as well as all sociodemographic, clinical, functional and psychological variables with p < .10 in univariate analysis. A forward- stepwise strategy was used. A value of p < .05 was considered significant for all analyses. We determined the area under the receiver operating characteristics (ROC) curve. The Statistical Package for Social Sciences 15.0 for Windows was used (SPSS Inc. USA).

## Results

A total of 208 patients (108 asthma and 100 COPD) were included in the study. The mean (SD) age of the patients with asthma was 51 (18) years (range: 16–84); 55% were female and 9% were current smokers. The mean (SD) disease duration was 18 (16.1) years and mean (SD) post-bronchodilatation forced expiratory volume in one second (FEV_1_) was 96.4 (21)% predicted. Asthma severity according to GINA criteria was: 2.8% mild intermittent, 18.5% mild persistent, 48% moderate persistent and 30.6% severe persistent.

The mean (SD) age of COPD patients was 66 (8.4) years (range: 40–80); 7% were female and 23% were current smokers. Mean disease duration was 9.2 (7.3) years and post-bronchodilatation FEV_1_ was 59.1 (19.5)% predicted; mean BODE index was 2.13 (2.01) and 6MWT was 383 (96.4) meters. COPD severity following the GOLD classification was 12% mild, 46% moderate, 32% severe and 10% very severe. Both groups of patients (asthma and COPD) differed in all sociodemographic, clinical and functional variables mentioned (p < .05).

A more detailed description of the baseline characteristics of the patients was included in a previously published article [21].

Differences in sociodemographic, clinical, functional and psychological variables between patients with and without exacerbations are shown in Table 1 for asthmatic patients and Table 2 for COPD patients.

**Table 1:** Sociodemographic, clinical, functional and psychological characteristics of asthmatic patients with and without ED visits or hospitalizations in the first and the second year of follow-up

	**1st year**				**2nd year**			
	**ED visits**		**Hospitalizations**		**ED visits**		**Hospitalizations**	
	**0**	**≥1**	**0**	**≥1**	**0**	**≥1**	**0**	**≥1**
	**n = 94**	**n = 14**	**n = 100**	**n = 8**	**n = 101**	**n = 7**	**n = 105**	**n = 3**
Sociodemographic variables								
Age, year	51.5 ± 18.3	50.4 ± 17.1	51.0 ± 18.4	56.5 ± 12.2	51.5 ± 18.4	49.1 ± 13.2	51.5 ± 17.9	46.3 ± 26.1
Female, n (%)	48 (51)	12 (86)*	54 (54)	6 (75)	54 (53)	6 (86)	58 (55)	2 (67)
Age at diagnosis, year	30.7 ± 21.7	28.1 ± 22.1	29.8 ± 21.8	37.0 ± 20.7	30.7 ± 22.0	24.8 ± 16.2	30.4 ± 21.6	26.6 ± 30.9
Disease duration, year	17.0 ± 15.6	22.5 ± 19.3	17.8 ± 16.2	16.7 ± 16.1	17.2 ± 16.1	24.3 ± 16.3	17.6 ± 16.4	20.0 ± 5.3
Active smoking, n (%)	8 (8.5)	2 (14)	10 (10)	0 (0)	9 (10)	1 (11)	10 (9)	0 (0)
*Mean pack-year*	12.3 ± 6.8	9 ± 7.4	11.9 ± 6.8	11.0 ± 12.7	11.8 ± 6.9	12.3 ± 9.3	12.0 ± 6.9	5.0 ± 0.1
Education level, n (%)								
*Illiterate/Primary School*	48 (51)	9 (64)	51 (51)	6 (75)	53 (52)	4 (57)	56 (53)	1 (33)
*Secondary School/University*	46 (49)	5 (36)	49 (49)	2 (25)	48 (48)	3 (43)	49 (47)	2 (67)
Socioeconomic level, n (%)								
*Low/Medium-low*	31 (33)	2 (14)	30 (30)	3 (37.5)	31 (31)	2 (29)	33 (31)	0 (0)
*Medium-medium/Medium-high/High*	63 (67)	12 (86)	70 (70)	5 (62.5)	70 (69)	5 (71)	72 (69)	3 (100)
Employment status, n (%)								
Working	30 (32)	3 (21)	32 (32)	1 (13)	31(31)	2 (29)	32 (30)	1 (33)
*Retired/Disability*	64 (68)	11 (79)	68 (68)	7 (87)	70 (69)	5 (71)	73 (70)	2 (67)
Place of residence, n (%)								
*Rural*	27 (29)	3 (21)	28 (28)	2 (25)	29 (29)	1 (14)	30 (28.5)	0 (0)
*Urban*	67 (71)	11 (79)	72 (72)	6 (75)	72 (71)	6 (86)	75 (71)	3 (100)
Marital status (%)								
*Single/separated/divorced/widowed*	31 (33)	4 (28)	34 (34)	1 (12.5)	33 (33)	2 (28)	34 (32)	1 (33)
*Married/unmarried partner*	63 (67)	10 (72)	66 (66)	7 (87.5)	68 (67)	5 (72)	71 (68)	2 (66)
Clinical-functional variables								
BMI, kg/m^2^ MRC dyspnea scale, n (%)	28.0 ± 4.8	27.9 ± 5.3	27.7 ± 4.8	31.2 ± 3.4*	27.9 ± 4.6	29.0 ± 7.7	28.0 ± 4.9	26.44 ± 2.6
*Grade 0-1-2*	82 (90)	9 (75)	86 (89)	5 (83)	86 (88)	5 (100)	88 (88)	3 (100)
*Grade 3-4*	9 (10)	3 (25)	11 (11)	1 (17)	12 (12)	0 (0)	12 (12)	0 (0)
Spirometry								
FVC post (%)	104.8 ± 17.8	106.1 ± 13.4	105.0 ± 17.1	105.8 ± 21.1	92.9 +0 .2	89.5 ± 21.2	92.0 ± 21.5	93.4 ± 25.8
FEV_1_ post (%)	95.6 ± 21.7	101.6 ± 15.5	96.1 ± 21.0	100.5 ± 22.0	97.0 + 21.1	87.0 + 19.0	96.5 + 21.1	82.3 + 21.0
GINA classification								
*GINA I-II*	21 (22)	2 (14)	23 (23)	0 (0)*	22 ( 22)	1 (14)	23 (22)	0 (0)
*GINA III-IV*	73 (78)	12 (86)	77 (77)	8 (100)	79 ( 78)	6 (86)	82 (78)	3 (100)
Previous year exacerbations								
Hospitalizations	0.2 ± 0.4	0.3 ± 0.6	0.2 ± 0.4	0.7 ± 0.5^Ŧ^	0.2 ± 0.4	0.4 ± 0.5	0.2 ± 0.4	1.3 ± 0.6 ^†^
ED visits	1.0 ± 1.4	1.8 ± 1.9	1.0 ± 1.4	2.4 ± 1.5 ^†^	1.1 ± 1.5	1.2 ± 1.7	1.1 ± 1.4	3.0 ± 2.0*
Psychological variables								
HADS anxiety	5.5 + 4.3	8.8 ± 3.3^†^	5.8 ± 4.3	3.5 ± 3.2**	3.3 ± 3.2	5.9 ± 5.9	3.8 ± 3.9	4.1 ± 5.3
HADS depression	3.4 ± 3.3	5.7 ± 3.3^†^	7.9 ± 3.4	6.5 ± 4.3*	3.6 ± 3.5	5.5 ± 4.7	3.9 ± 3.7	4.5 ± 4.8*

Data are presented as mean ± standard deviation (X ± SD), unless otherwise indicated.

*BMI* body mass index, *MRC scale* Medical Research Council dyspnea scale, *FVC post* forced vital capacity post-bronchodilatation, *FEV*_*1*_*post* forced expiratory volumen in 1-s post-bronchodilatation, *GINA* Global Initiative for Asthma.

P values: comparisons between groups were tested using the Pearson´s X^2^ or Fisher´s Exact test (categorical variables) or ANOVA (normally distributed continuous variables) and Mann–Whitney U test (non- normally distributed variables or non-parametric data).

** p < .1 *p < .05; †p < .01; Ŧp < .001.

**Table 2:** Sociodemographic, clinical, functional and psychological characteristics of COPD patients with and without ED visits or hospitalizations in the first and the second year of follow-up

	**1st year**				**2nd year**			
	**ED visits**		**Hospitalizations**		**ED visits**		**Hospitalizations**	
	**0**	**≥1**	**0**	**≥1**	**0**	**≥1**	**0**	**≥1**
	**n = 79**	**n = 21**	**n = 87**	**n = 13**	**n = 79**	**n = 21**	**n = 81**	**n = 19**
Sociodemographic variables								
Age, year	66.0 ± 7.9	65.5 ± 10.2	65.3 ± 8.7	70.1 ± 5.0	65.2 ± 8.8	68.7 ± 6.1	65.3 ± 8.6	69.0 ± 6.8
Female, n (%)	5 (6)	2 (9)	7 (8)	0 (0)	6 (7)	1 (5)	6 (7)	1 (5)
Age at diagnosis, year	56.8 ± 9.1	55.4 ± 7.5	56.7 ± 8.8	55.8 ± 8.3	56.1 ± 8.7	58.5 ± 9.0	56.3 ± 8.8	57.7 ± 8.7
Disease duration, year	9.1 ± 7.3	9.6 ± 7.7	8.4 ± 6.8	14.3 ± 8.8^*^	9.0 ± 6.9	10.1 ± 8.8	8.8 ± 7.0	10.8 ± 8.5
Active smoking, n (%)	15(19)	8 (38)**	20(23)	3(23)	19(24)	4(19)	17 (22)	5 (26)
*Mean pack-year*	63.0 ± 25.1	59.6 ± 27.3	62.9 ± 25.1	58.1 ± 28.3	63.2 ± 2.2	59.1 + 27.0	61.1 ± 24.6	67.4 ± 29.2
Education level, n (%)								
*Illiterate/Primary School*	52 (66)	16 (76)	57 (66)	11 (85)	52 (66)	16 (76)	52 (64)	16 (84)
*Secondary School/University*	27 (34)	5 (24)	30 (34)	2 (15)	27 (34)	5 (24)	29 (36)	3 (16)
Socioeconomic level, n (%)								
*Low/Medium-low*	31 (39)	13 (62) **	36 (41)	8 (61)**	34 (43)	10 (48)	34 (42)	10 (53)
*Medium-medium/Medium-high/High*	48 (61)	8 (38)	51 (59)	5 (39)	45 (57)	11 (52)	47 (58)	9 (47)
Employment status, n (%)								
Working	11 (14)	4 (19)	15 (17)	0 (0)	14 (18)	1 (5)	15 (18)	0
*Retired/Disability*	68 (86)	17 (81)	72 (83)	13 (100)	65 (82)	20 (95)	66 (81)	19 (100)^*^
Place of residence, n (%)								
*Rural*	21 (27)	5 (24)	24 (28)	2 (15)	23 (29)	3 (14)	21 (26)	5 (26)
*Urban*	58 (73)	16 (76)	63 (72)	11 (85)	56 (71)	18 (86)	60 (74)	14 (74)
Marital status n (%)								
*Married/unmarried partner*	65 (82)	18 (86)	73 (84)	10 (77)	66 (83)	17 (81)	67 (83)	16 (84)
*Single/separated/divorced/widowed*	14 (18)	3 (14)	14 (16)	3 (23)	13 (16)	4 (19)	14 (17)	3 (16)
Clinical-functional variables								
BMI, kg/m^2^	28.4 ± 4.6	26.0 ± 4.1**	28.2 ± 4.5	25.4 ± 4.4**	28.3 ± 4.6	26.1 ± 4.3	28.1 ± 4.6	26.7 ± 4.7
MRC dyspnea scale, n (%)								
*Grade 0-1-2*	68 (86)	15 (72)	74 (85)	12 (92)	66 (84)	17 (81)	69 (85)	14 (74)
*Grade 3-4*	11 (14)	6(28)	13 (15)	1 (8)	13 (16)	4 (19)	12 (15)	5 (26)
Spirometry								
FVC post (%)	92.9 ± 2.0	89.5 ± 21.2	92.0 ± 21.5	93.4 ± 25.8	91.6 ± 21.2	94.7 ± 25.2	93.7 ± 22.5	85.7 ± 18.2
FEV_1_ post (%)	59.5 ± 19.3	57.5 ± 20.5	59.9 ± 18.8	54.0 ± 23.6	59.3 ± 18.3	58.2 ± 24.3	60.8 ± 19.6	51.7 ± 17.6
6MWT, m	394.2 ± 86.5	341.1 ± 120.7**	395.1 ± 83.6	302.3 ± 136.3^†^	393.1 ± 88.4	345.1 ± 117.0	396.8 ± 83.8	324.1 ± 124.3^†^
BODE index (0–10)	1.9 ± 1.9	2.8 ± 2.5	1.87 ± 1.8	3.8 ± 2.5^*^	2.0 ± 1.8	2.5 ± 2.7	1.9 ± 1.8	3.2 ± 2.4^*^
GOLD classification								
GOLD 1-2	46 (58)	12 (57)	53 (61)	5 (38)	48 (61)	10 (48)	49 (60)	9 (47)
GOLD 3-4	33 (42)	9 (43)	34 (39)	8 (62)	31 (39)	11 (52)	32 (40)	10 (53)
Hospitalizations previous year	0.3 ± 0.7	1.0 ± 1.8^*^	0.25 ± 0.6	1.6 ± 2.1^Ŧ^	0.3 ± 0.6	1.0 ± 1.8^†^	0.2 ± 0.5	1.5 ± 1.9^Ŧ^
ED visits previous year	1.0 ± 1.6	0 ± 1.7	1.0 ± 1.5	2.2 ± 3.2	1.2 ± 1.9	1.2 ± 1.9	1.0 ± 1.5	1.9 ± 2.8
Psychological variables								
HADS anxiety	3.3 ± 3.2	5.9 ± 5.9	3.8 ± 3.9	4.1 ± 5.3	4.0 ± 3.9	3.3 ± 4.5	4.0 ± 4.1	3.3 ± 4.1
HADS depression	3.6 ± 3.5	5.5 ± 47	3.9 ± 3.7	4.5 ± 4.8	4.0 ± 3.9	3.8 ± 3.9	3.9 ± 3.7	4.3 ± 4.4

Data are presented as mean ± standard deviation (X ± SD), unless otherwise indicated.

*BMI* body mass index, *MRC scale* Medical Research Council dyspnea scale, *FVC post* forced vital capacity post-bronchodilatation, *FEV1 post* forced expiratory volumen in 1-s post-bronchodilatation, *6MWT* 6-min walking test, *BODE index* Body mass index, Degree of Airflow Obstruction, Dyspnea and Exercise Capacity Index; *GOLD* Global Initiative for Chronic Obstructive Lung Disease.

P values: comparisons between groups were tested using the Pearson´s X^2^or Fisher´s Exact test (categorical variables) or ANOVA (normally distributed continuous variables) and Mann–Whitney U test (non-normally distributed variables or non-parametric data).

** p < .1 *p < .05; †p < .01; Ŧp < .001.

Differences in the scores of the HRQoL questionnaires between patients with and without exacerbations are shown in Tables 3 and 4 for asthma and COPD, respectively. Asthmatic patients with ED visits in the first year of follow-up showed statistically significant differences in the AQ20, the AQLQ total and subscales scores, and the SGRQ total and subscales scores, except Symptoms, in comparison with patients without ED visits. Asthmatic patients with hospitalizations in the first year of follow-up also showed worse scores in the AQ20 and in the SGRQ total and subscales scores, except Symptoms. In the second year, patients with ED visits only showed significant differences in the score of the SGRQ Impact subscale and patients with hospitalizations, in the score of the SGRQ Symptoms subscale.

**Table 3:** Baseline scores on HRQoL questionnaires of patients with and without ED visits or hospitalizations due to asthma in the first and the second year of follow-up

**HRQoL questionnaires**	**1st year**				**2nd year**			
	**ED visits**		**Hospitalizations**		**ED visits**		**Hospitalizations**	
	**0**	**≥1**	**0**	**≥1**	**0**	**≥1**	**0**	**≥1**
	**n = 94**	**n = 14**	**n = 100**	**n = 8**	**n = 101**	**n = 7**	**n = 105**	**n = 3**
AQ20	7.3 ± 4.6	11.6 ± 5.4^†^	7.5 ± 4.7	12.4 ± 5.1^*^	7.7 ± 4.8	10.3 ± 6.2	7.8 ± 4.8	11.6 ± 6.4
SGRQ scores								
Total	29.8 ± 17.9	43.1 ± 14.7^†^	30.1 ± 17.6	48.9 ± 14.9^†^	30.6 ± 17.9	44.5 ± 17.0	31.0 ± 17.9	48.7 ± 16.3
Symptoms	41.4 ± 20.3	43.5 ± 22.0	40.7 ± 20.4	53.8 ± 17.9	41.9 ± 20.7	38.3 ± 16.5	40.8 ± 19.9	70.1 ± 16.2*
Activity	37.2 ± 24.7	55.1 ± 19.6^*^	37.8 ± 24.2	60.9 ± 22.4^*^	38.3 ± 24.5	57.2 ± 23.5	39.1 ± 24.8	54.3 ± 23.3
Impact	21.9 ± 18.0	36.2 ± 15.7^†^	22.4 ± 17.8	40.5 ± 16.6^†^	22.7 ± 17.9	39.3 ± 18.1*	23.4 ± 18.2	38.9 ± 15.5
AQLQ scores								
Total	5.2 ± 0.9	4.3 ± 1.0^Ŧ^	5.2 ± 0.9	4.7 ± 1.0	5.1 ± 0.9	4.9 ± 1.2	5.1 ± 0.9	4.6 ± 1.2
Symptoms	5.6 ± 1.2	4.7 ± 1.1^*^	5.5 ± 1.2	5.0 ± 1.0	5.5 ± 1.2	5.2 ± 0.9	5.5 ± 1.2	5.2 ± 0.8
Activities	4.6 ± 0.9	3.8 ± 1.0^†^	4.5 ± 0.9	4.1 ± 1.0	4.5 ± 0.9	4.5 ± 1.4	4.5 ± 0.9	3.8 ± 1.2
Emotional functioning	5.7 ± 1.2	4.7 ± 1.4^†^	5.6 ± 1.3	5.5 ± 1.1	5.0 ± 1.3	5.3 ± 1.8	5.6 ± 1.3	4.8 ± 2.2
Environmental stimuli	5.6 ± 1.3	4.2 ± 1.7^†^	5.5 ± 1.4	4.7 ± 1.5	5.5 ± 1.4	4.8 ± 1.8	5.4 ± 1.4	5.0 ± 1.7

Data are presented as mean ± standard deviation (X ± SD).

*AQ20* Airways Questionnaire 20, *SGRQ* St George´s Respiratory Questionnaire, *AQLQ* Asthma Quality of Life Questionnaire.

P values: comparisons between groups were tested using t test Mann–Whitney U (non-parametric data).

** p < .1 *p < .05; †p < .01; Ŧp < .001.

**Table 4:** Baseline scores on HRQoL questionnaires of patients with and without ED visits or hospitalizations due to COPD in the first and the second year of follow-up

**HRQoL questionnaires**	**1st year**				**2nd year**			
	**ED visits**		**Hospitalizations**		**ED visits**		**Hospitalizations**	
	**No**	**≥1**	**No**	**≥1**	**No**	**≥1**	**No**	**≥1**
	**n = 79**	**n = 21**	**n = 87**	**n = 13**	**n = 79**	**n = 21**	**n = 81**	**n = 19**
AQ20	7.0 ± 4.1	8.4 ± 4.1	7.3 ± 4.3	7.4 ± 2.8	7.4 ± 4.3	6.9 ± 3.3	7.3 ± 4.4	7.0 ± 3.0
CCQ								
Total	1.5 ± 0.9	2.1 ± 1.2^*^	1.5 ± 1.0	2.2 ± 1.0*	1.6 ± 1.0	1.7 ± 1.0	1.5 ± 1.0	1.8 ± 1.0
Symptoms	1.8 ± 1.2	2.4 ± 1.4	1.9 ± 1.2	2.4 ± 1.2	2.0 ± 1.3	2.0 ± 1.2	1.9 ± 1.1	2.0 ± 1.2
Functional	1.2 ± 1.1	1.7 ± 1.3	1.2 ± 1.1	2.0 ± 1.1*	1.2 ± 1.1	1.5 ± 1.3	1.4 ± 1.3	1.8 ± 1.3
Mental	1.3 ± 1.4	2.2 ± 1.5*	1.4 ± 1.4	2.1 ± 1.5	1.4 ± 1.4	1.6 ± 1.8	1.6 ± 1.6	1.9 ± 1.4
SGRQ scores								
Total	36.7 ± 18.4	42.6 ± 16.8	36.5 ± 18.0	47.7 ± 16.1*	37.6 ± 18.5	39.3 ± 17.0	36.8 ± 18.4	42.8 ± 16.4
Symptoms	38.8 ± 18.4	41.6 ± 20.9	38.2 ± 18.2	47.5 ± 21.8	38.7 ± 18.1	42.0 ± 21.9	38.8 ± 18.2	42.1 ± 21.8
Activity	50.7 ± 23.5	56.9 ± 19.4	50.1 ± 23.0	64.8 ± 16.2*	51.2 ± 23.3	54.9 ± 20.7	49.8 ± 23.1	61.1 ± 19.0
Impact	28.1 ± 19.9	34.7 ± 18.6	28.2 ± 19.9	38.1 ± 16.4*	29.5 ± 20.3	29.5 ± 17.6	28.8 ± 20.5	32.6 ± 16.3
CRQ scores								
Total	98.3 ± 16.2	87.0 ± 23.2	97.1 ± 18.3	87.6 ± 18.8	96.9 ± 18.0	91.7 ± 20.2	96.2 ± 18.8	93.7 ± 17.8
Dyspnea	19.1 ± 4.7	17.8 ± 5.1	18.8 ± 4.9	18.5 ± 4.1	18.9 ± 5.0	18.5 ± 4.3	18.7 ± 5.0	19.0 ± 4.2
Fatigue	19.5 ± 4.5	17.2 ± 5.9	19.4 ± 4.8	16.5 ± 5.2	19.3 ± 4.7	17.8 ± 5.6	19.2 ± 4.8	17.8 ± 5.6
Emotional Function	37.4 ± 7.4	32.2 ± 11.6	36.6 ± 8.6	34.0 ± 9.9	36.5 ± 8.5	35.2 ± 9.8	36.2 ± 8.9	36.4 ± 8.5
Mastery	22.6 ± 5.0	19.8 ± 7.2	22.5 ± 5.4	18.5 ± 6.0*	22.4 ± 5.2	20.2 ± 6.8	22.3 ± 5.5	20.4 ± 6.3

Data are presented as mean ± standard deviation (X ± SD).

*AQ20* Airways Questionnaire 20, *CCQ* Clinical COPD Questionnaire (CCQ), *SGRQ* St George´s Respiratory Questionnaire, *CRQ*: Chronic Respiratory Disease Questionnaire.

P values: comparison between groups were tested using t test Mann–Whitney U (non-parametric data).

*p < .05; †p < .01; Ŧp < .001.

In COPD, only the CCQ Total and Mental domain showed significant differences between patients with and without ED visits in the first year. As regards to hospitalizations in the first year, differences appeared in the CCQ Total and Functional subscale, the SGRQ Total and all subscales scores, except Symptoms, and the CRQ Mastery subscale. During the second year of follow-up, no differences were found in the HRQoL questionnaires between patients with and without exacerbations.

Predictive models of ED visits and hospitalizations in the first year of follow-up in asthma and COPD patients for those HRQoL questionnaires that showed differences in both groups of patients (zero ED visits or hospitalizations versus ≥ 1 ED visits or hospitalizations) adjusted by sociodemographic, clinical, functional and psychological variables are shown in Table 5.

**Table 5:** Logistic regression analyses predicting ED visits and hospitalizations of asthma and COPD patients in the first year of follow-up

	**ED visits**				**Hospitalizations**			
	**Predictors**	**OR**	**95% CI**	**p**	Predictors*	**OR**	**95% CI**	**p**
**ASTHMA**								
AQ20 Model								
	AQ20	1.19	1.05-1.34	0.004	AQ20	1.21	1.01-1.45	0.041
					BMI	1.25	1.01-1.54	0.034
					Previous hosp.	12.98	2.57-65.49	0.002
SGRQ Model								
	HADS anxiety	1.18	1.04-1.34	0.01	BMI	1.23	0.99-1.51	0.034
					Previous hosp.	10.07	2.19-46.41	0.003
AQLQ Model								
	AQLQ total score	0.97	0.45-0.99	0.002	-	-	-	-
					BMI	1.27	1.04-1.54	0.016
					Previous hosp.	11.12	2.56-48.24	0.001
**COPD**								
CCQ Model	CCQ total score	1.06	1.00-1.11	0.036	-	-	-	-
	BMI	0.87	0.76-0.99	0.041	BMI	0.79	0.66-0.96	0.002
	Previous hosp.	1.79	1.12-2.87	0.014	Previous hosp.	2.60	1.44-4.69	0.001
SGRQ Model								
	-	-	-	-	BMI	0.82	0.68-0.98	0.03
					Previous hosp.	2.55	1.42-4.60	0.001
					Disease duration	1.09	1.00-1.18	0.03

*Only predictors with p values < .0.5 are included.

*AQ20* Airways Questionnaire 20, *HADS* Hospital Anxiety and Depresion Scale, *CCQ* Clinical COPD Questionnaire, *BMI* Body mass index.

Variables with p < .1 included in the different multivariate models are: 1) ED visits in asthma: age, sex, HADS anxiety, HADS depresion 2) Hospitalizations in asthma: age, sex, GINA severity , previous ED visits , previous hospitalizations, BMI, HADS anxiety, HADS depresion. 3) ED visits in COPD: age, sex, previous hospitalizations, BMI, socioeconomical level, active smoking, 6MWT. 4) COPD hospitalizations: age, sex, disease duration, BMI, socioeconomical level, 6MWT, BODE index, previous hospitalizations.

In the AQ20 model for asthmatic patients, the AQ20 total score emerged as an independent predictor for ED visits (OR: 1.19; p = .004) and hospitalizations (OR: 1.21; p = .04) in the first year of follow-up. In the SGRQ model, the questionnaire lost significance after adjusting for anxiety, which emerged as the only predictor of ED visits (OR: 1.18; p = .01). In the AQLQ model, the total score emerged as predictor of ED visits (OR: 0.9; p = .002), but it did not predict hospitalizations.

In COPD patients, the CCQ emerged as an independent predictor of ED visits in the first year of follow-up (OR: 1.06; p = .036), but it did not predict hospitalizations. In the SGRQ and the CRQ models, none of these questionnaires emerged as independent predictors of COPD exacerbations in the first year.

Among the considered sociodemographic, clinical and functional variables, the BMI and the frequency of previous hospitalizations were predictors of hospitalizations in the first-year of follow-up in all models, and the disease duration emerged also as predictor in the SGRQ model, both in asthma and COPD patients. In the CCQ model, the BMI and the frequency of previous hospitalizations predicted emergency visits in COPD.

In the second year of follow-up, none of the assessed HRQoL questionnaires were independent predictors of exacerbations, except the Impact domain of the SGRQ, which emerged as independent predictor of ED visits due to asthma (OR: 1.04; p = .03).

The frequency of previous hospitalizations was the only independent predictor of hospitalizations in the second year, both in asthma (OR: 2.30; p = .006) and in COPD (OR: 3.01; p = 0.007). The comparison between the HRQoL questionnaires that emerged as predictors of exacerbations in asthmatic patients revealed that the AQ20 had predictive ability for ED visits in the first year (area under the ROC curve 0.723) similar to longer questionnaires such as the AQLQ (area under the ROC curve 0.750). Area under the ROC curve for prediction of hospitalizations was 0.759 for the AQ20. In COPD, area under the ROC curve for the CCQ predicting ED visits in the first year was 0.651.

## Discussion

Our results show that brief HRQoL questionnaires such as the AQ20 and the CCQ are independent predictors of the use of healthcare resources. The AQ20 predicts ED visits and hospitalizations in asthma and the CCQ predicts ED visits in COPD. The predictive value of both questionnaires has proven to be as high as of the longer disease-specific questionnaires such as the SGRQ, the AQLQ and the CRQ. The predictive ability of the AQ20 and the CCQ is only shown in the first year of follow-up, suggesting the convenience of a periodic administration, at least annually.

In asthma, the AQ20 showed an ability to predict ED visits in the first year similarly to the AQLQ and a higher predictive value to predict hospitalizations. To our knowledge, no studies comparing the ability of AQ20 and AQLQ to predict exacerbations have been carried out. In comparison with the SGRQ, the AQ20 shows higher ability to predict both ED visits and hospitalizations in the first year. To date, there is only one study [40] comparing the ability of the AQ20 and the SGRQ to predict exacerbations retrospectively, and it showed that both the AQ20 score (OR 1.15, 95% CI 1.05 to 1.25) and the SGRQ score (OR 1.03, 95% CI 1.01 to 1.06) were significantly associated with exacerbations experienced in the previous six months. This finding agrees with the present study suggesting that the AQ20 could be a good alternative to the SGRQ.

In COPD, the AQ20 did not emerge as a significant predictor of ED visits or hospitalizations in the two-year of follow-up. This aspect has not been previously addressed. In the Spanish validation study of the questionnaire [21] we also observed better psychometric properties in asthma than in COPD, suggesting that, although it can be used in patients with several respiratory diseases, it shows higher applicability in asthmatic patients.

In COPD, the CCQ emerged as an independent predictor of ED visits in the first year, with a higher predictive value than the CRQ and the SGRQ, as neither of them emerged as predictors. There are no data in literature analyzing the CRQ ability to predict exacerbations. Although in earlier literature the SGRQ was associated with higher frequency of exacerbations and admissions [3,10,19], these studies included more severe COPD patients and did not adjust for HADS anxiety-depression, except for one study [10]. To date, there is only one study [41] that has shown that impairment in the CCQ in weekly sequential measurements allows for early identification of exacerbations (defined as the increase of symptoms registered in a diary), but it did not consider ED visits or hospitalizations and it did not compare the CCQ with other questionnaires.

The predictive ability of the CCQ reported in the present study has not been identified before and our results are potentially relevant to help in the selection of short HRQoL questionnaires in clinical practice.

It should also be noted that none of the assessed HRQoL questionnaires were found to be predictors of exacerbations in the second year of follow-up, except the SGRQ Impact subscale, which emerged as predictor of ED visits due to asthma. Amongst the assessed sociodemographic, clinical and functional variables, only BMI and hospitalizations in the previous year emerged as independent predictors of ED visits in the first year of follow-up in COPD, and also predicted hospitalizations in the first year in both asthma and COPD. In asthma, our data agree with Rodrigo et al. [42], who found that patients with BMI > 25 kg/m2 showed significant increases in length of ED stay and rate of hospitalization, despite adjustments for other confounding variables. In COPD, our findings differ from other authors who found that the independent predictive ability of BMI disappeared in models including dyspnea [12,43]. Possibly, measuring dyspnea with the Shortness of Breath Questionnaire instead of the MRC scale used in our study could explain the difference between the obtained results. However, our results agree with previous investigations which pointed out prior exacerbations as the most important predictor of future exacerbations, both in asthma [44,45] and in COPD [3,9]. These results support recent observations in literature concerning the presence of an exacerbating phenotype independent of functional severity [46].

It is important to emphasize that studies aimed to identify predictors of exacerbations in asthma and COPD often disregard the psychological variables [10]. However, it is widely known that anxiety and depression are related to exacerbations [9,10,43] and HRQoL [10,18,19] in both diseases.

In asthma, our results agree with previous studies showing that ED visits in the first year of follow-up are more frequent in patients with worse scores in anxiety and depression [8], although in the multivariate analysis HADS loses significance after adjusting for HRQoL measured with the AQ20 or the AQLQ. Nevertheless, in the model for the SGRQ, anxiety remained as an independent predictor of ED visits due to asthma (instead of SGRQ). These results probably suggest that the emotional dimension is not well captured by the SGRQ. In fact, the SGRQ Impact subscale is heterogeneous and does not just consider emotional aspects (in our sample, Cronbach’s alpha was 0.47).

In COPD, we did not find differences in terms of anxiety and depression scores in patients with ED visits or hospitalizations, whereas other authors [10] have shown that anxiety emerged as a predictor of readmissions, probably due to the higher functional severity in their patients and their higher scores in the HADS. Furthermore, only 7% of COPD patients of our sample were female and other authors reported higher anxiety and depression scores in women versus men [47] and, consequently, a higher association to hospital readmissions [48].

The results of this study must be interpreted taking into account several limitations. Firstly, we did not assess the level of asthma control, which is one of the factors shown to predict the use of health care resources [49]. Secondly, in COPD we could have included the COPD Assessment Test (CAT) [50] in the comparison because it has been recently proposed by GOLD strategy [51]. The role of both questionnaires (CAT and CCQ) in optimizing everyday care of COPD has been recently analyzed [52]. In the present study, we used the CCQ because the questionnaire has some advantages over the CAT, such as the division in domains, the preference manifested by the patients [53], and its excellent psychometric properties including the determination of the minimal clinically important difference (MCID) calculated by several methods [54,55]. The CCQ is included in the 2013 GOLD update [56]. Thirdly, the sample size was small and inclusion of stable patients without comorbidities resulted in a low exacerbation rate. In fact, a very limited number of patients required hospitalization especially in the asthma group. In COPD, the sample mainly comprised males, possibly due to the smoking habits in Spanish males and females in the age group of our cohort [57]. This could have affected generalizability of our results to women. Lastly, we have not taken into account those exacerbations not severe enough to require medical attention, although it is known that they can have a negative impact on quality of life [58]. Our study population makes it possible to minimize the misclassification of patients with comorbid disease such as congestive heart failure, allowing us to focus primarily on predictors related to respiratory diseases. This could also explain the low mortality rate in the cohort.

Further investigations in other populations with comorbidities are needed in order to confirm whether the simplified and short HRQoL questionnaires (AQ20 and CCQ) can contribute (in addition to prior exacerbations) to identifying, in routine practice, a subgroup of patients more prone to future exacerbations, in order to take preventive measures for their avoidance or early management.

To our knowledge, this report is the first to simultaneously compare the ability of several short HRQoL instruments applicable in routine care and longer instruments used in research to predict exacerbations in both types of airway diseases (stable asthma and COPD patients). Our findings allow us to conclude that the AQ20 is a better predictor in asthma and the CCQ, in COPD.

In conclusion, the AQ20 in asthma and the CCQ in COPD showed similar or even higher ability to predict exacerbations than longer disease-specific questionnaires. Therefore, these brief and easy to complete questionnaires allow for introducing the HRQoL measurement in the routine clinical practice in a periodic pattern, in order to identify patients with higher risk of exacerbations.

## Competing interest

The authors declare that they have no competing interests.

## Authors' contributions

MBA, IV, HVH participated in the design of the study, coordination, implementation, analysis and interpretation of the data and wrote the manuscript. SPF and SPD participated in the analysis and interpretation of the data. All authors read and approved the final manuscript.
